# Ergonomics of laparoscopic graspers and the importance of haptic feedback: the surgeons’ perspective

**DOI:** 10.1007/s10397-016-0959-z

**Published:** 2016-06-04

**Authors:** Chantal C. J. Alleblas, Michel P. H. Vleugels, Theodoor E. Nieboer

**Affiliations:** 1 Department of Obstetrics and Gynecology, Radboud University Medical Center, P.O. Box 9101, 6500HB Nijmegen, The Netherlands; 2 Department of Obstetrics and Gynecology, Riverland Hospital, Tiel, The Netherlands

**Keywords:** Laparoscopy, Ergonomics, Human-product interaction, Haptic feedback

## Abstract

Haptic feedback is drastically reduced in laparoscopic surgery compared to open surgery. Introducing enhanced haptic feedback in laparoscopic instruments might well improve surgical safety and efficiency. In the design process of a laparoscopic grasper with enhanced haptic feedback, handle design should be addressed to strive for optimal usability and comfort. Additionally, the surgeons’ perspective on the potential benefits of haptic feedback should be assessed to ascertain the clinical interest of enhanced haptic feedback. A questionnaire was designed to determine surgeons’ use and preferences for laparoscopic instruments and expectations about enhanced haptic feedback. Surgeons were also asked whether they experience physical complaints related to laparoscopic instruments. The questionnaire was distributed to a group of laparoscopic surgeons based in Europe. From the 279 contacted subjects, 98 completed the questionnaire (response rate 35 %). Of all respondents, 77 % reported physical complaints directly attributable to the use of laparoscopic instruments. No evident similarity in the main preference for graspers was found, either with or without haptic feedback. According to respondents, the added value of haptic feedback could be of particular use in feeling differences in tissue consistencies, feeling the applied pressure, locating a tumor or enlarged lymph node, feeling arterial pulse, and limiting strain in the surgeon’s hand. This study stresses that the high prevalence of physical complaints directly related to laparoscopic instruments among laparoscopic surgeons is still relevant. Furthermore, the potential benefits of enhanced haptic feedback in laparoscopic surgery are recognized by laparoscopic specialists. Therefore, haptic feedback is considered an unmet need in laparoscopy.

## Background

In laparoscopic surgery, haptic feedback should enable surgeons to perceive interaction forces between instrument and tissue. This is beneficial information regarding accurate regulation of tissue manipulation forces and recognition of tissue characteristics. In open surgery, the surgeon is able to manipulate tissue directly with the gloved hand; i.e., the surgeon directly perceives haptic feedback. In contrast, during laparoscopy, the surgeon can only manipulate tissue indirectly due to the interference of instruments, which are inserted through small incisions. Consequently, haptic feedback is drastically reduced in laparoscopic surgery compared to open abdominal surgery. This is mainly caused by the friction within instruments and dynamic properties of the laparoscopic surgical setup [1, 2]. Introducing enhanced haptic feedback in laparoscopic instruments might well be beneficial for surgical safety and efficiency.

The results of several (pre)clinical studies show that haptic feedback is deficient in laparoscopic surgery [3–5]. Moreover, intra-operative complications appear to be often the result of intentional actions, resulting in unintentional outcomes, caused by visual misperception [6–8]. Additionally, surgical specialists have identified technology as one of the most important risk domains for patient safety [9]. Tholey et al. found that the availability of both visual and haptic feedback leads to better tissue characterization than exclusively visual or haptic feedback [10]. Previous studies argue for the implementation of enhanced haptic feedback to increase efficiency in terms of more successful grasping actions [11] and accurate control over the instrument-tissue interaction forces [12]. Two recently published literature reviews provide an overview of studies that have been performed regarding haptic feedback in minimally invasive surgery [2, 13]. The authors conclude that both patients and surgeons may well benefit from enhanced haptic feedback in minimally invasive surgical equipment. Although several technological efforts have been made in artificial settings, it is argued that a clinically driven approach should be deployed for a feasible application in surgical practice [14].

Laparoscopic instruments are known to cause physical discomfort [15, 16] and, moreover, to cause injuries especially affecting the thumbs [17, 18]. Furthermore, almost all laparoscopic handles come with the adage “one size fits all” whereas small hand size is a known risk factor for experiencing physical discomfort and difficulties in the use of laparoscopic instruments [19–21]. Instrument handles are the most important physical interface for laparoscopic surgeons [22]. To strive for optimal usability and comfort, handle design should be specifically addressed during the design process of new types of surgical instruments.

Related to the development of a laparoscopic haptic feedback grasper [23], the tools that are already used in laparoscopy need to be evaluated. The involvement of end users in the design process is indispensible for suitability, safety, and acceptance [24, 25]. Therefore, the aim of this study was to perform an evaluation of expert opinions regarding handle designs of currently used laparoscopic gaspers and to determine surgeons’ needs and expectations regarding haptic feedback instruments.

## Methods

A questionnaire was designed to determine the surgeons’ current use of instruments, their physical complaints related to instrument use, as well as their needs and preferences for laparoscopic instruments. Furthermore, we aimed to identify expectations regarding haptic feedback in future instrument developments. The survey was distributed among attendees of the 23rd annual congress of the European Society for Gynecological Endoscopy (September 2014) and the annual meeting of the Dutch Working Group for Gynecological Endoscopy (October 2014). Additionally, an online version was distributed among the members of the Dutch Society of Endoscopic Surgery (January 2015). The questionnaire was accompanied with an explanation of the aim and was subdivided into categories concerning demographics, physical complaints related to laparoscopic instrument use, handgrip assessment of currently used laparoscopic graspers, preferences for handle designs, and expectations regarding implementation of haptic feedback in laparoscopic surgery. Questions and answer options are presented in the Appendix. A descriptive data analysis was performed with SPSS software, version 22.

## Findings

### Demographics

A total of 279 subjects were contacted. The number of returned questionnaires was 98 (response rate 35 %), among which were 63 gynecologists, 27 general surgeons, 4 urologists, 2 pediatric surgeons, and 2 medical technicians. The majority of respondents were male (68 %). Four respondents were left-handed, and 9 respondents were ambidextrous. All respondents worked in Europe of which the majority was established in The Netherlands (86 %). Table 1 presents the additional demographic data.

**Table 1:** Demographic information

Characteristics	Data	
	Mean	Standard deviation
Age in years	45.5	8.9
Glove size (general)	7.4	0.6
Glove size (men)	7.6	0.4
Glove size (women)	6.8	0.4
Years of experience	17.7	8.5
Years of experience in endoscopy	13.5	8.2
Endoscopic procedures per month	16.5	14.2

### Physical complaints

**Fig. 1:**
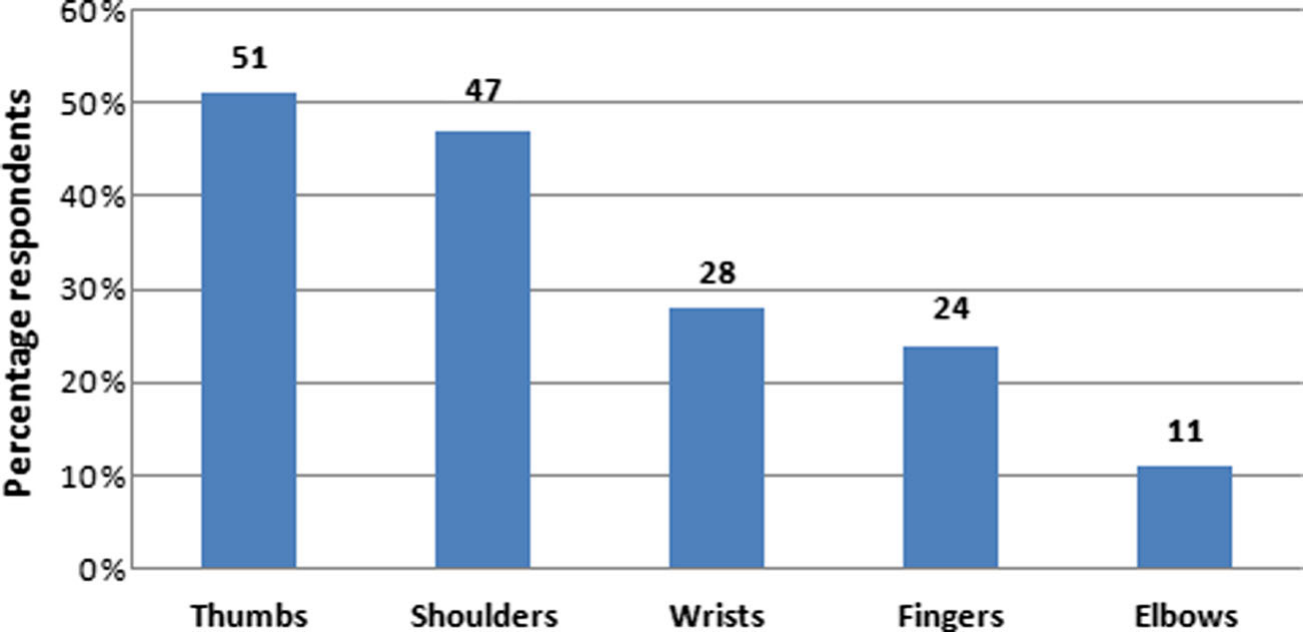
Prevalence of physical complaints in the upper extremities (directly attributable to the use of laparoscopic instruments)

**Fig. 2:**
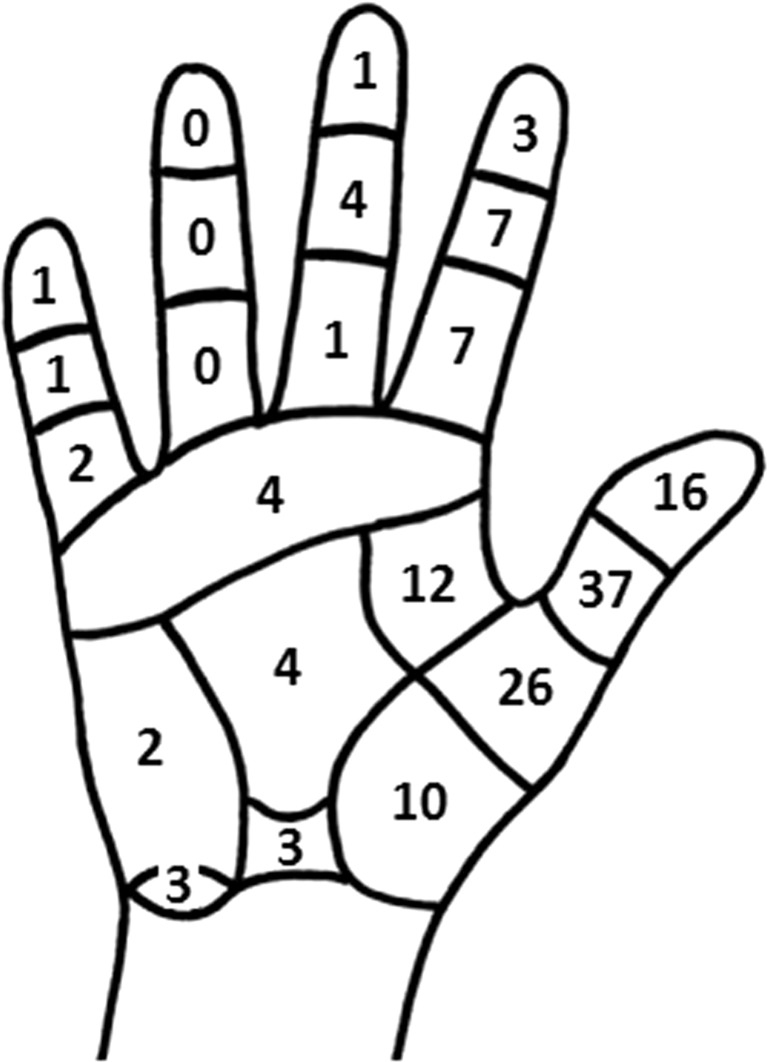
Hand map [26] including the frequency of reported areas of discomfort due to pressure caused by instruments

Overall, 77 % of the surgeons reported physical complaints directly attributable to the use of laparoscopic instruments. Figure 1 illustrates the prevalence of physical complaints as indicated for specific parts of the upper extremities. The frequency of discomfort in the palm of the hand from pressure caused by instruments as indicated by the surgeons is illustrated in Fig. 2 [26].

### Handgrip assessment

**Fig. 3:**
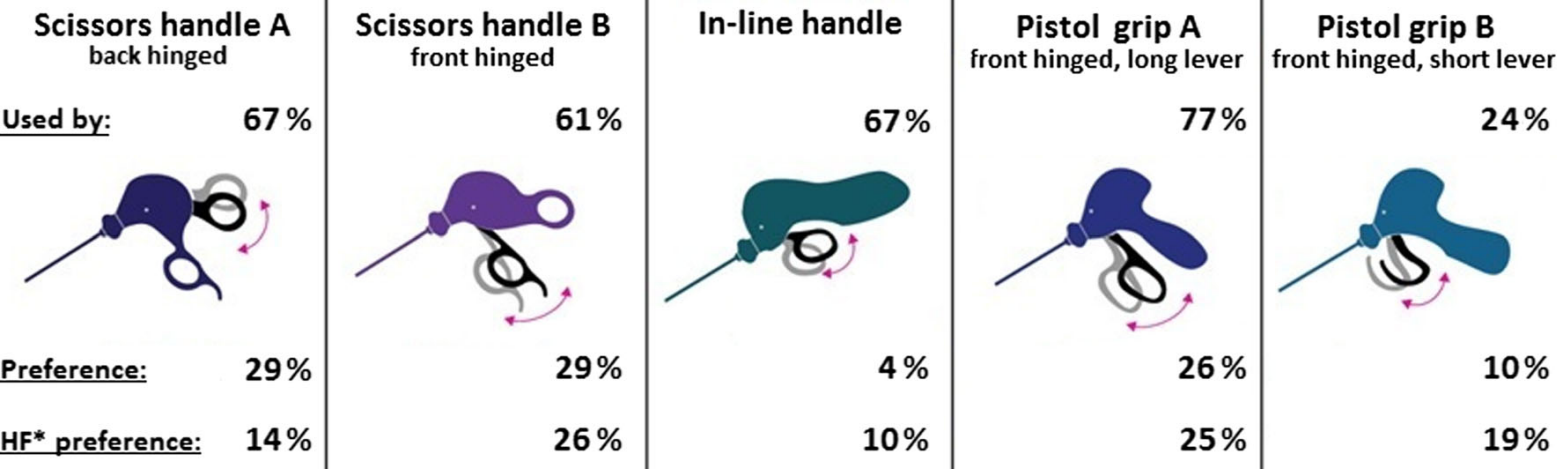
Presented handles for assessment including use and preferences for current use and future haptic feedback instruments. *HF* haptic feedback

Handles including indicated use and preferences by respondents are shown in Fig. 3. The long-lever pistol grip was most commonly used. When combined, 99 % of respondents indicated that they used at least one of the two types of scissors handles. Respondents were asked in what percentage of laparoscopic procedures they used each handle type. A total of 24 % respondents indicated that they used the back-hinged scissors handle during all procedures. For the front-hinged scissors handle, this was 32 %. Less often used as standard equipment was the in-line handle (4 %) and the long-lever pistol grip (12 %), whereas the short-lever pistol grip was never reported to be used in all procedures. When specifically asked what kind of handle would be preferred for a haptic feedback instrument, the front-hinged scissors handle and the long-lever pistol grip were most frequently chosen. Regarding the usability of handgrips, three aspects including functionality, comfort, and freedom of movement were assessed on a 7-point Likert scale. The long-lever pistol grip scored the highest on all the three aspects (Table 2).

**Table 2:** Handgrip usability assessment

Handle	Functionality	Comfort	Freedom of movement
Scissors handle A	4.4 ± 1.8	3.8 ± 1.7	4.1 ± 1.5
Scissors handle B	5.0 ± 1.4	4.6 ± 1.4	4.9 ± 1.3
In-line handle	4.0 ± 1.6	4.6 ± 1.5	4.7 ± 1.6
Pistol grip A	5.3 ± 1.4	5.3 ± 1.4	5.0 ± 1.3
Pistol grip B	4.5 ± 1.8	4.6 ± 1.7	4.4 + 1.6

For illustrations of the handle types, see Fig. 3. Assessment was based on a 7-point Likert scale where 1 means “the worst” and 7 means “the best” for the constructs’ functionality and freedom of movement. Comfort was assessed on a 7-point Likert scale where 1 means “very uncomfortable” and 7 means “very comfortable”

**Fig. 4:**
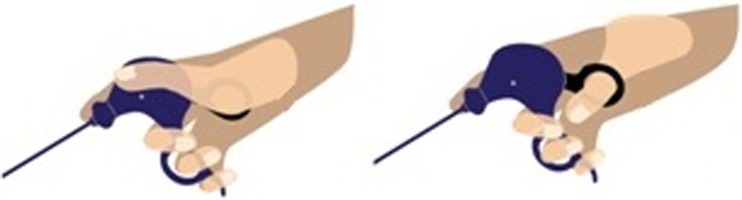
Illustration of the palm grip (*left*) versus the usual grip (*right*)

Two extra user features were evaluated. Respondents were asked to estimate what percentage of time they positioned their index finger forward on the rotation knob of the handle. The majority (48 %) of respondents reported to adopt this grip during less than a quarter of the overall procedure time, and 16 % reported to adopt this grip for over 75 % of the procedure time. Furthermore, 51 % of respondents indicated to control a scissors handle by means of a so-called “palm grip” as illustrated in Fig. 4. The most frequently reported reasons to do this were as follows: in case of more static surgical steps, in case the application of more force is necessary, or in order to relieve strain or pressure on the thumb.

### Haptic feedback

To estimate the added value of haptic feedback in clinical scenarios, respondents were asked to assess nine scenarios on a 6-point Likert scale where 0 means “not useful” and 5 means “very useful” for clinical practice. The results are presented in Table 3. The possibility to feel differences in tissue consistencies and the ability to feel how much pressure is being applied were expected to be the most promising outcomes of integrated haptic feedback. Reduction of operation time and reduction of conversions to open surgery were least expected be a consequence of enhanced haptic feedback.

**Table 3:** Assessment of the utility of haptic feedback in clinical scenarios

Scenario	Mean ± SD^a^
Feeling differences in tissue consistencies	3.5 ± 1.5
Locating a tumor or enlarged lymph node	3.2 ± 1.7
Feeling arterial pulse	2.7 ± 1.6
Feeling how much pressure is being applied	3.6 ± 1.4
Limiting the force on the surgeons’ hand	3.4 ± 1.5
Lowering the time to complete surgery	2.4 ± 1.7
Reducing complications	3.2 ± 1.6
Reduction of conversions to open surgery	2.1 ± 1.6
Performing laparoscopy instead of open surgery	2.4 ± 1.7

^a^Assessment based on a 6-point Likert scale ranging from 0 to 5 and presented as mean ± SD

## Discussion

In this study, expert experiences and opinions regarding handle designs of laparoscopic graspers and regarding implementation of enhanced haptic feedback were evaluated. This study shows, with a prevalence of 77 %, that physical complaints related to the use of laparoscopic instruments are commonly experienced. Whereas direct questioning revealed no similar handgrip preference among the surgical specialists, the handgrip usability assessment results favored the long-lever pistol grip design. Furthermore, the results regarding the utility assessment of haptic feedback show clinical support for the implementation of enhanced haptic feedback in laparoscopic graspers.

Exposure to risk factors for developing physical complaints should obviously be avoided. In the context of laparoscopic instrument use, these risk factors involve adverse postures and motions of the upper extremities, adverse force exertion and excessive local pressure, or friction in the contact surface between instrument and hand [27]. Other risk factors, including precise working and repetitive movements, are apparently inherent to tasks that are to be performed during laparoscopic surgery. However, these factors can also be reinforced by suboptimal surgical instrument design [11].

Respondents did not show evident similarity in their main preference for graspers, either with or without haptic feedback. However, the long-lever pistol grip was best appraised in the usability assessment. Fifty-one percent of the respondents do sometimes control a scissors handle by means of a so-called palm grip, which approaches the hand posture when controlling a pistol grip. Moreover, our results emphasize that discomfort as a result of contact pressure is frequently experienced in the thumb and thenar area. Based on the indicated use of instruments, we concluded that this pressure-induced discomfort is a result from the use of scissors handles. Additionally, two recent studies also reported clinical support for a pistol-grip handle design. A pistol grip would specifically meet the need to alleviate contact stress during instrument control [28, 29]. In summary, these results suggest that a haptic feedback grasper is best equipped with a pistol grip.

As mentioned in the “Background” section, laparoscopic handles usually come with the adage “one size fits all.” A laparoscopic stapler generally comes with a long-lever pistol grip. Sutton et al. reported that the handles of these devices are too big for a certain group of surgeons, particularly women, who have significantly smaller hands than men [19]. Therefore, two or more sizes should be considered to ascertain suitability for the whole range of end users.

The potential benefits which haptic feedback yields are acknowledged by the respondents. More specifically, according to laparoscopic specialists, enhanced haptic feedback could be of particular use in feeling differences in tissue consistencies, feeling how much pressure is being applied, locating a tumor or enlarged lymph node, feeling arterial pulse, and enhanced instrument ergonomics in terms of limiting the force on the surgeons’ hand.

This study provides directives for the handle design of a haptic feedback grasper. As suggested by Matern et al. during the design process of surgical instruments, muscle activity and task performance under dynamic conditions should be considered [30]. Based on the results of the questionnaire and the principles of haptic feedback, we may hypothesize that haptic feedback is an unmet need in laparoscopic surgery. Along with the development of such a device, the assessed scenarios should be examined in (pre-)clinical experimental research.

Rather than a direct assessment of readily available instruments, this assessment was based on pictures which can be considered as a limitation of our study. A large group of respondents report to use a front-hinged scissors handle, whereas the vast majority of scissors handles used are equipped with back-hinged actuation. We might consider this an artifact of the used method, but we might as well question whether surgeons are aware of the actuation of the instrument. Lastly, since the vast majority of respondents were Dutch, we have to be reticent to extrapolate these findings to Europe as a whole.

## Conclusion

This study highlights the clinical importance of well-designed ergonomic laparoscopic instruments. Moreover, the need of haptic feedback in laparoscopic surgery is recognized by surgeons of different disciplines. Both patients and surgeons may well benefit from the implementation of enhanced haptic feedback in laparoscopic instruments.

## Appendix

**Table 4:** Survey questions

Demographics	
What is your age?	N
What is your gender?	S
In which country do you work?	S
In what department do you work?	S
What is your dominant hand?	S
What is your surgical glove size?	N
For how many years are you in practice?	N
For how many years do you perform laparoscopic surgery?	N
How many laparoscopic procedures do you perform per month?	N
Physical symptoms	
Have you ever experienced physical complaints or discomfort that you would attribute to the use of laparoscopic instruments?	S
If applicable, in which parts of the upper extremities have you experienced these physical complaints or discomforts?	M
If applicable, where do you experience discomfort from pressure caused by instruments? (includes the hand map as illustrated in Fig. 2)	M
Handgrip assessment	
In what percentage of laparoscopic procedures do you use these handle types?	N
Which handle type is your favorite for grasping?	S
How do you rate the functionality of each handle type for grasping tasks?	L
How do you rate the comfort of each handle type for grasping tasks?	L
How do you rate the freedom of movement of each handle type for grasping tasks?	L
Which handle type would be your favorite for grasping with an instrument that provides enhanced haptic feedback?	S
User features	
When holding a laparoscopic grasper with rotating function for the instrument tip, what percentage of time do you keep your index finger pointed forward?	N
Do you sometimes “palm” your grip when operating with a scissors handle?	S
If so, in what situations do you do this?	D
Clinical relevance	
If you had a laparoscopic tool with haptic feedback, for what specific scenarios would you consider it useful?	L
- Feeling differences in tissue consistencies	
- Locating a tumor or enlarged lymph node	
- Feeling arterial pulse	
- Feeling how much pressure is being applied	
- Limiting the force on the surgeons’ hand	
- Lowering the time to complete surgery	
- Reducing complications	
- Reduction of conversions to open surgery	
- Performing laparoscopy instead of open surgery	

Body parts involved the following: wrists, fingers, thumbs, elbows, and shoulders. Handgrip assessments concerned the evaluation of the following designs: back-hinged scissors handle, front-hinged scissors handle, in-line handle, long-lever pistol grip, and short-lever pistol grip

*D* descriptive, *L* Likert scale, *M* multiple answers, *N* numeric response, *S* single answer
